# Effect of Wheat Bran on Fecal Butyrate-Producing Bacteria and Wheat Bran Combined with Barley on *Bacteroides* Abundance in Japanese Healthy Adults

**DOI:** 10.3390/nu10121980

**Published:** 2018-12-14

**Authors:** Seiichiro Aoe, Fumiko Nakamura, Suguru Fujiwara

**Affiliations:** 1 Department of Food Science, Faculty of Home Economics, Otsuma Women’s University, 12 Sanban-cho, Chiyoda-ku, Tokyo 102-8357, Japan; 2 CPCC Company Limited, 3-3-5 Uchikanda, Chiyoda-ku, Tokyo 101-0047, Japan; f.nakamura@cpcc.co.jp (F.N.); s.fujiwara@cpcc.co.jp (S.F.)

**Keywords:** wheat bran, BARLEYmax, fecal butyrate, butyrate producer, *Bacteroides*

## Abstract

Wheat bran (WB) is rich in insoluble arabinoxylan, while BARLEYmax (BM) is a barley line that is rich in fructan, resistant starch, and β-glucan. In the present study, we investigated which of these two fiber sources would produce more favorable changes in the fecal variables of healthy subjects. Sixty healthy subjects were randomly divided into four groups (*n* = 15 per group) and fed twice daily for 4 weeks with baked cereal bars containing neither WB nor BM (WB−BM−), WB without BM (WB+BM−), BM without WB (WB−BM+), or WB and BM (WB+BM+). At baseline and after 4 weeks, the fecal microbiota composition and the concentrations of short-chain fatty acids were measured. A significant interactive effect of WB and BM on the abundance of genus *Bacteroides* was observed at week 4. The abundance of butyrate-producing bacteria and the fecal concentration of *n*-butyrate were significantly higher in the WB+ groups than in the WB− groups. In conclusion, WB was associated with elevated fecal concentrations of short-chain fatty acids including butyrate owing to an increase in the abundance of butyrate-producing bacteria. Additionally, the combination of WB and BM was associated with an increase in the abundance of genus *Bacteroides*. Therefore, both WB alone and WB combined with BM favorably influenced the fecal variables of healthy subjects.

## 1. Introduction

Epidemiological studies have reported that the consumption of whole grain cereals or cereal brans may reduce intestinal transit time, increase the bacterial fermentation of fiber to short-chain fatty acids (SCFAs) with anticarcinogenic properties, and thereby reduce the risk of colorectal cancer [1,2,3]. A recent systematic review concluded that high-fiber, wheat-based cereals can improve bowel function with grade A evidence (i.e., can be trusted to guide clinical practice) [4].

The mechanisms through which fiber can improve bowel function remain to be fully elucidated. One possible mechanism proposed by Sonnenburg et al. [5] is that microbiota-accessible carbohydrates found in dietary fiber may substantially influence the microbial ecosystem in the gut. They showed that a diet low in such carbohydrates resulted in a progressive loss of microbiota diversity, which is a type of dysbiosis. Other possible forms of dysbiosis include (i) an expansion of pathobionts; (ii) a change in the microbial composition, i.e., an increase or reduction in the abundance of an indicator species; and (iii) a change in microbial functional capacity [6]. It is considered that a diet high in fat and low in fermentable dietary fiber shifts the microbiota toward a more dysbiotic pattern associated with an increased risk of intestinal inflammation [7].

Wheat bran (WB) is a promising fiber source that is rich in insoluble fiber (35.0–48.4 g/100 g) [8] and arabinoxylan (22–30 g/100 g), which is an almost-insoluble fiber linked with ferulic acid [9]. A systematic review concluded that insoluble fiber (WB) showed favorable results for the treatment of irritable bowel disease [10]. It has also been reported that WB induced a significant increase in fecal bulk, a reduction in intestinal transit time, and a significant increase in the frequency of bowel movements in healthy people [11]. The structures of WB and the aleurone fraction of wheat have rarely been mentioned or characterized in interventional studies [12]. The structure of a food product containing these fractions can markedly affect the bioavailability of bioactive compounds and nutrients, thus influencing their effects on metabolism and health. The modification of WB and aleurone fractions by baking can influence their effects on the colon by altering the fermentation of their dietary fiber components. SCFAs such as acetate, propionate, and butyrate are important metabolites of microbial fermentation, and butyrate has been suggested to potentially exert effects on health [9]. Recent reports have shown that arabinoxylan in WB is the main component responsible for improving various aspects of intestinal barrier function with the involvement of compositional changes of the microbiota [13]. Additionally, the provision of amylase-pretreated WB as the sole added energy source to human intestinal microbial communities in anaerobic fermentors led to an increase in the abundance of butyrate producers [14].

In addition, soluble fiber-rich barley is expected to reduce the risk of dysbiosis. Barley is rich in β-glucan, which is easily fermented by *Bacteroides* and *Prevotella* [15]. BARLEYmax (*Tantangara*; BM) is a barley line developed by CSIRO (Commonwealth Scientific and Industrial Research Organisation) in Australia. BM is not a genetically modified crop and it is rich in fructan and resistant starch as well as β-glucan. A recent report showed that subjects with an intake of 12 g/day of BM showed significant increases in fecal output and defecation frequency [16]. Compared to the parameters at baseline, an intake of 12 g/day of BM resulted in a significant increase in the production of SCFAs, an increase in the abundance of *Bacteroides* and a decrease in the abundance of *Clostridium subcluster XIVa* [16].

The combination of multiple types of dietary fiber that have different fermentation rates has recently been a topic of interest. It has been reported that rats fed a diet containing resistant starch together with WB had a greater fecal excretion of SCFAs than those fed resistant starch or WB alone, suggesting that the incorporation of WB delayed the site of fermentation of the resistant starch to the distal part of the hindgut [17]. It was concluded that the combination of indigestible carbohydrates may affect both the profile of SCFAs produced by fermentation and the site of SCFA release in the rat hindgut. Concordantly, a human intervention study showed that combining WB with resistant starch had more benefits than supplementation with WB alone [18]. 

In the present study, we investigated the effects of dietary supplementation with WB and BM individually or in combination in healthy Japanese adult volunteers. We evaluated which dietary fiber source would produce more favorable changes in fecal variables (e.g., higher butyrate, lower phenols, and lower ammonia). We also investigated whether combining WB with BM would modify the fecal microbiota more favorably than supplementation with WB or BM alone.

## 2. Materials and Methods 

### 2.1. Study Design

**Figure 1:**
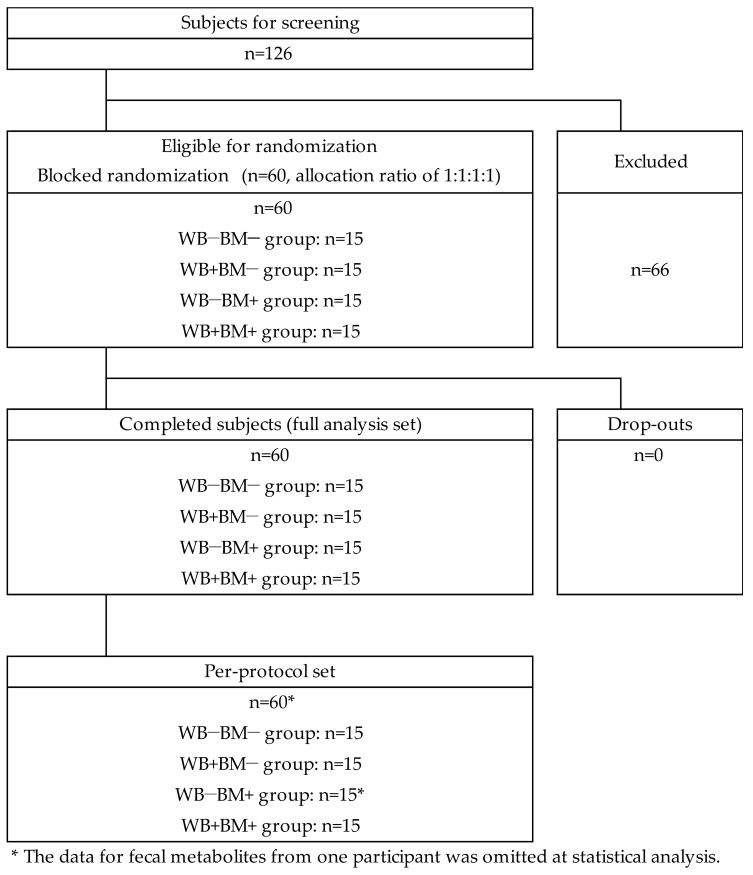
Scheme of participant enrollment.

The study was designed as a double-blind controlled randomized trial. Subjects consumed one of four test foods twice a day for 4 weeks as a supplement to their staple foods. The scheme of participant enrollment is shown in Figure 1. The study was performed in conformity with the regulations of the Institutional Review Board of Chiyoda Paramedical Care Clinic (IRB No. 15000088) and the Declaration of Helsinki. All the subjects gave written informed consent before entering the study. The study protocol was registered at the University Hospital Medical Information Network Clinical Trials Registry (UMIN 000027569).

### 2.2. Subjects

We enrolled ethnically Japanese subjects who were recruited by CPCC (Creative Pioneer for Clinical Challenge) Co., Ltd. (Tokyo, Japan). The inclusion criteria were as follows: aged 20–64 years, a defecation frequency of less than 5 times/week (i.e., showing a tendency towards constipation) [19], a low intake of fiber (lower than 12 g/day according to simple dietary records for 4 days comprising 3 weekdays and 1 weekend day), an understanding of the study procedures, and provided written informed consent to participate. 

The exclusion criteria were as follows:

1. Regular use of a medicine for intestinal disorders or an aperient (including a laxative).
2. Regular consumption of health foods thought to improve constipation at the time of the screening examination.
3. Use of any drugs that could affect digestion and nutrient absorption including antibiotics at the time of the screening examination.
4. Inability or unwillingness to stop consuming probiotic or prebiotic supplements such as lactic acid bacteria, *Bifidobacterium*, *Bacillus subtilis* var. natto, food fortified with oligosaccharides, dietary fiber, large quantities of sugar or alcohol, barley-rich food, or other dietary health supplements for the entire duration of the study.
5. Having a food allergy.
6. Having a disease urgently needing treatment or having serious complications.
7. Having a history of a digestive organ disease or an operation that could affect digestion, nutrient absorption, or bowel movements.
8. Judged from the answers to the subject background questionnaire to be inappropriate as a subject.
9. Judged from the results of a blood test during the screening examination to be inappropriate as a subject.
10. Currently pregnant or intended to become pregnant during the study period.
11. Currently nursing an infant.
12. Having any drug dependency.
13. Having an anamnesis or medical history of alcohol dependence.
14. Participating in another study involving the intake of food, drugs, or cosmetics, or judged by one of the examining doctors involved in the trial to be inappropriate as a subject, e.g., subjects who plan to change their lifestyle during the test period or who would not obey the rules of the trial.

Sixty subjects participated in this trial, were stratified four blocks according to their sex and age. Fifteen subjects were assigned to each trail group by blocked randomization. (WB−BM−, WB−BM+, WB+BM−, and WB+BM+). The subjects ate self-selected foods as accompanying dishes along with the test foods and otherwise maintained the same eating habits before and throughout the trial. Anthropometric measurements (body weight, height, and waist circumference) were performed, and blood samples were collected from a forearm vein and analyzed at baseline and after 4 weeks. Table 1 summarizes the baseline characteristics of the subjects. The four groups were similar with regard to their age, body weight, body mass index, body fat, and blood pressure. 

### 2.3. Dietary Supplementation

**Figure 2:**
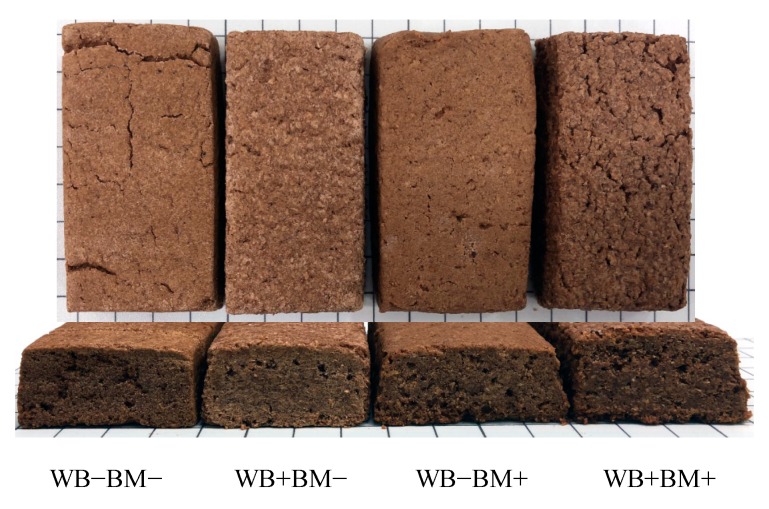
Features of the test foods.

The four test foods comprised baked cereal bars composed as follows: (1) neither WB nor BM (WB−BM−), (2) WB without BM (WB+BM− diet), (3) BM without WB (WB−BM+ diet), and (4) WB and BM (WB+BM+ diet). The subjects ate the cereal bars (40.7 g) twice daily as a supplement to their staple food. The test foods were manufactured by Ayabe-Yougashi Corp. (Saitama, Japan). First, margarine and brown sugar were mixed together, then egg and vanilla extract were added and all the ingredients were mixed together. Wheat flour, wheat bran, crushed BM (mesh size 2.07 mm), and cellulose were added in the proportions shown in Table 1 and mixed. The dough was kept at 4 °C for 120 min, then sheeted to 15 mm thickness and cut into 40 × 75 mm bar shapes (approximately 46 g/bar). The bars were baked with a tunnel oven at the upper temperature of 146 °C and lower temperature of 135 °C for 40 min. Then, the bars were cooled to room temperature and individually pillow-packed with aluminum metallized film. The components of the test foods are listed in Table 2. Wheat flour was obtained from Nitto Fuji Flour Milling Co., Ltd. (Tokyo, Japan). WB (K-Bran) was obtained from Hoshino Bussan Co. Ltd. (Gunma, Japan), cellulose (Ceolus FD-301) was obtained from Asahi Kasei Corp. (Tokyo, Japan), and BM (BARLEYmax) was obtained from Teijin Limited (Tokyo, Japan). Pearled BM was used for the test foods. The contents of total, soluble, and insoluble dietary fiber in the samples were analyzed using the method of Prosky et al. (AOAC 991.43) [20]. The β-glucan content in the test foods was measured using the method of McCleary et al. (AOAC 995.16) [21]. The resistant starch content in each sample was analyzed using the method of AOAC 2002.02 [22]. The arabinoxylan content in the test food was calculated from the contents in the WB and BM measured using a previously described method [23]. The nutrient components were analyzed by Japan Food Research Laboratories (Tokyo, Japan) and are listed in Table 3. The average weight of the test foods was 40.7 g and their caloric contents were almost equal. Their total dietary fiber contents were almost equal adjusted by cellulose. We formulated the test foods to taste good by incorporating cocoa powder and brown sugar, and it was difficult to detect a difference in taste among the test foods. As a result, blindness was properly maintained. The features of the test foods are shown in Figure 2. The biggest difference among the test foods was the source of dietary fiber.

### 2.4. Food Frequency Questionnaire

Daily food intake records were investigated via interviews in which the subjects described their recent meals. A 4-day record of their diet was completed by a registered dietitian at baseline and after 4 weeks. Food records were analyzed using the Standard Tables of Food Composition in Japan 2015, Seventh Revised Version [24] and the software program Excel Eiyokun Version 8 (Kenpakusha, Tokyo, Japan). 

### 2.5. Fecal Collection 

Fecal samples were collected at baseline and after 4 weeks from 60 healthy adult volunteers at the Chiyoda Paramedical Care Clinic, Tokyo, Japan, using fecal collection tubes (TechnoSuruga Laboratory Co., Ltd., Shizuoka, Japan). Samples were immediately frozen at −20 °C, transported on dry ice, and then stored at −80 °C until further analysis.

### 2.6. Anthropometrical Measures and Biochemical Analysis

Body mass index using an InBody570 body composition analyzer (InBody Japan, Inc., Tokyo, Japan). Physical examination (body weight, body mass index, body fat) were conducted using an InBody570 body composition analyzer (InBody Japan, Inc., Tokyo, Japan).

Vital signs (systolic blood pressure, diastolic blood pressure, pulse rate) were measured by fully automatic blood pressure monitor TM2656VPW (A&D Company, Ltd., Tokyo, Japan).

Blood samples and urine samples were obtained after overnight fast for clinical examinations performed by a commercial laboratory (LSI Medience Corporation, Tokyo, Japan). In particular, serum proteins (total protein (TP), albumin (ALB)), serum enzymes (aspartate aminotransferase (AST), alanine transaminase (ALT), lactate dehydrogenase (LDH), alkaline phosphatase (ALP), gamma-glutamyl transpeptidase (γ-GTP), creatine phosphokinase (CPK)), serum lipid (total cholesterol (T-Cho), HDL cholesterol (HDL-Cho), LDL cholesterol (LDL-Cho), triglyceride(TG)), serum total bilirubin (T-BIL), serum urea nitrogen (BUN), serum creatinine (CRE), serum uric acid (UA), serum electrolyte (Na, Cl, K, Ca), blood fasting glucose (GLC) and glycosylated albumin (HbA1c), hematological examinations (white blood cell (WBC), red blood cell (RBC), hemoglobin (Hb), hematocrit (Ht), platelet (PLT)), and urinary qualitative tests were conducted. These markers were analyzed to examine the safety of the test foods. Specifically, serum protein, T-BIL, and the several enzymes measured are markers of liver function, serum lipids are markers of lipid metabolism, serum BUN, CRE, UA, and electrolytes are markers of kidney function, and blood GLC and HbA1c are markers of glucose metabolism. Hematological examinations (WBC, RBC, Hb, Ht, PLT) are markers of inflammation, anemia, and coagulation abnormality.

### 2.7. DNA Extraction and Analysis of Intestinal Microbiota Composition by High-Throughput Sequencing

Each fecal sample (about 100 mg) was suspended in GTC buffer (Tris-HCl (pH 9.0), Tris-ethylenediaminetetraacetic acid (pH 8.0), guanidine thiocyanate, and bromothymol blue). After centrifugation, DNA was extracted from the suspension using an automatic nucleic acid extractor (Precision System Science, Chiba, Japan). MagDEA DNA 200 (GC) (Precision System Science) was used as the reagent for automatic nucleic acid extraction [25]. 

The V3–V4 region of the bacterial 16S rRNA gene was amplified from the DNA samples using the 341f [26] and R806 [27] primers. The forward and reverse primers both contained an 8-bp indexing sequence to allow for multiplexing [25]. The V3–V4 regions of the bacterial 16S rRNA fragments were amplified and the PCR (polymerase chain reaction) products were purified through a MultiScreen™ PCRu96 filter plate (Merck Millipore, Burlington, MA, USA). To prepare the amplicon pool, the purified PCR products were quantified by real-time quantitative PCR (q-PCR) on a Rotor-Gene^®^ Q quantitative thermal cycler using MightyAmp^®^ real-time PCR buffer containing SYBR^®^ Plus (TaKaRa Bio, Inc., Shiga, Japan) [25]. The pooled PCR products were sequenced using the MiSeq^®^ reagent kit v.3 (Illumina, San Diego, CA, USA) via paired-end 2 × 284-bp cycles on the MiSeq^®^ system (600 cycles). The paired-end reads were concatenated using fastq-join with the default options [28]. Only joined reads that had a quality value score of ≥20 for more than 99% of the sequence were extracted using the fastq_quality_filter and fastq_quality_trimmer algorithms of the FASTX-Toolkit [29]. The chimeric sequences were deleted with usearch6.1 by using split-libraries-fastq.py in QIIME ver 1.8.0 (the University of Colorado, Colorado, USA) [30]. 

Bacterial identification from chimeric-filtered sequences was performed using the Ribosomal Database Project version 2.11 (Michigan State University, Michigan, USA) [31,32] and QIIME ver 1.8.0 pipeline [30]. The bacterial taxonomy identified was visualized using the Metagenome@KIN version 2.2.1 analysis software (cut off phylum > 0.8) (World Fusion, Tokyo, Japan). In QIIME, the sequences were clustered into operational taxonomic units (OTUs) on the basis of having >97% similarity with sequences in the Greengenes database [33] using pick_open_reference_otus.py in QIIME. The OTUs were tabulated on the taxonomic levels from phylum to genus and their relative abundances were calculated using plot_taxa_summary.py in QIIME. Plots showing the alpha diversity from open-reference picked OTU tables were constructed from the diversity metrics of phylogenetic diversity whole tree, chao1 index, observed OTU number (observed species), and Shannon index using alpha_diversity.py in QIIME. Beta diversity was calculated from the unweighted and weighted unifrac distance matrixes in beta_diversity.py in QIIME. All the above procedures were performed by TechnoSuruga Laboratory, Co., Ltd.

### 2.8. Fecal Organic Acids, Indoles, Phenols, and Ammonia

For the determination of organic acids, feces were suspended in distilled water, heated at 85 °C for 15 min to inactivate viruses, and then centrifuged according to the previous report [34]. It was confirmed that SCFAs are stable in feces at 85 °C by a previous study, in which the recovery rate of all SCFAs heated at that temperature was more than 96%. The concentrations of organic acids such as formic acid, acetic acid, propionic acid, isobutyrate, butyric acid, valeric acid, isovaleric acid, lactic acid, and succinic acid in feces were measured using a high-performance liquid chromatography organic acid analysis system with a Prominence CDD-10A conductivity detector (Shimadzu, Kyoto, Japan); two tandemly-arranged Shim-pack SCR-102(H) columns (300 mm × 8 mm inner diameter (ID)); a Shim-pack SCR-102(H) guard column (50 mm × 6 mm ID) [35]. The HPLC calibration curve for the measurement for the organic acids was done with prepared standards solutions.

For the measurement of indoles and phenols, feces were suspended with phosphate buffer including 4-isopropylphenol as an internal standard. Each sample was heated and extracted with acetonitrile and centrifuged. The supernatant was dehydrated and purified using a sodium sulfate drying cartridge (Bond Elut LRC, Agilent Technologies, Santa Clara, CA, USA) with a C18 cartridge (Smart SPE C18-30, AiSTI SCIENCE, Wakayama, Japan) and a PSA (primary-secondary amine) cartridge (Smart SPE PSA-30, AiSTI SCIENCE, Japan), and placed into a vial. Indoles and phenols in feces were measured using gas chromatography with mass spectrometry (QP-2010, Shimadzu) and a capillary column (inert cap WAX, 30 m × 0.25 mm × 0.25 µm, GL Sciences, Tokyo, Japan). The GC/MS (gas chromatography - mass spectrometry)calibration curve for the measurement for the indoles and phenols was done with prepared standard solutions.

For the measurement of the ammonium ion concentration, feces were suspended, filtered, and diluted in distilled water. The ammonium ion concentration in feces was measured using an ion chromatography system (ICS-1000, Dionex, Sunnyvale, CA, USA) with an IonPac™ CS12A column (4 mm × 250 mm, Dionex) and an IonPac™ CG12A guard column (4 mm × 50 mm, Dionex). 

All the above procedures were performed by TechnoSuruga Laboratory, Co., Ltd.

### 2.9. Statistical Analysis

Sample sizes were calculated from previous studies [16,36]. Fifteen subjects were required per group (type I error (α) = 0.05, 1 − β = 0.80). Data normality was checked using a quantile–quantile plot. Bartlett’s test was used to test for the homogeneity of variances. Data were analyzed using SPSS Statistics version 20 (IBM Corporation, Armonk, NY, USA). Two-way analysis of variance was applied for the primary (microbiota abundance) and secondary (fecal variables) outcomes. The Tukey–Kramer and Steel–Dwass multiple comparison tests were used to compare the values of all outcomes and nutritional intakes. A correlation analysis between fecal metabolites and bacterial abundance was performed using Spearman’s rank sum test. In all analyses, a two-sided *p*-value < 0.05 was considered significant. 

## 3. Results

### 3.1. Dietary Intake and Adherence

All the subjects completed the trial. No adverse events corresponding to the intake of the test food, such as gastrointestinal problems, were observed during the trial. Statistical analyses were performed for the subjects in each group (*n* = 15 per group) in the per-protocol set analysis.

Daily energy and nutrient intakes are summarized in Table 4. There were no significant differences among the calorie or nutrient intakes of the four groups during 4 weeks. The main dietary fiber sources of the subjects were vegetables, pulses, cereals, and potatoes. No noticeable difference in dietary fiber source among the groups was observed.

### 3.2. Anthropometrical Measures and Biochemical Analysis

The effects of WB and BM intakes on body weight, body mass index, body fat, and blood pressure at baseline and week 4 are shown in supplementary Table S1. No significant differences were detected among groups at baseline and week 4. The serum biochemistry results of the patients at baseline and week 4 are shown in supplementary Tables S2 and S3. At baseline, the mean serum concentration of albumin (ALB) in the WB+BM− group was significantly lower than that in the other three groups. However, this difference was quite small (0.2 g/dL). All the patients were included for this analysis. No other significant differences were detected among groups at baseline.

Slight but significant changes in alanine aminotransferase (ALT), lactate dehydrogenase (LDH), creatinine (CRE), and HbA1c (NGSP) were observed at week 4 as compared to baseline. These changes were random, were not observed in the specific test group, and were considered to be within the normal range of physiological variation. Urinary markers were normal. Therefore, it was considered that the intake of WB and BM individually or in combination had no harmful effects on the health of all subjects.

### 3.3. Fecal Microbiota Composition

**Figure 3:**
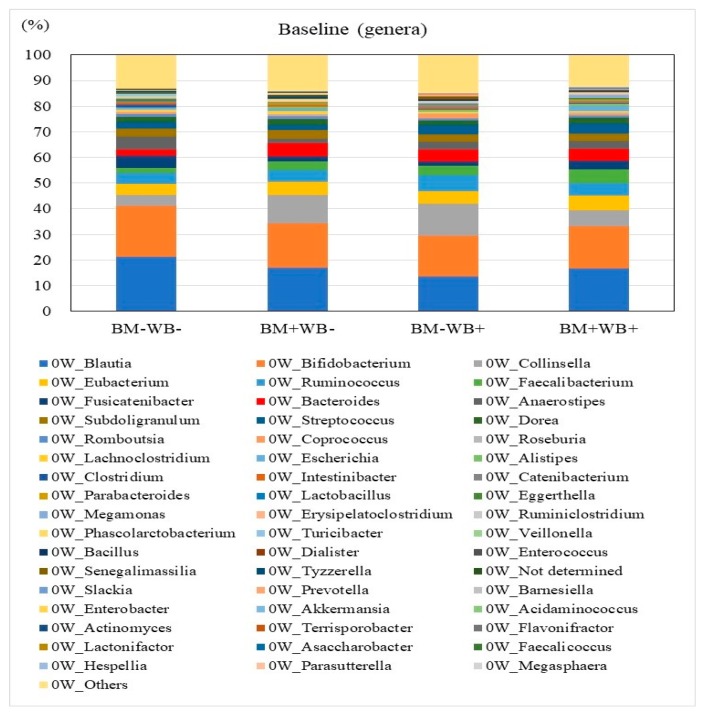
The average relative abundances of detected bacteria genera in stool samples obtained at baseline. No significant differences were observed among the test groups at baseline.

**Figure 4:**
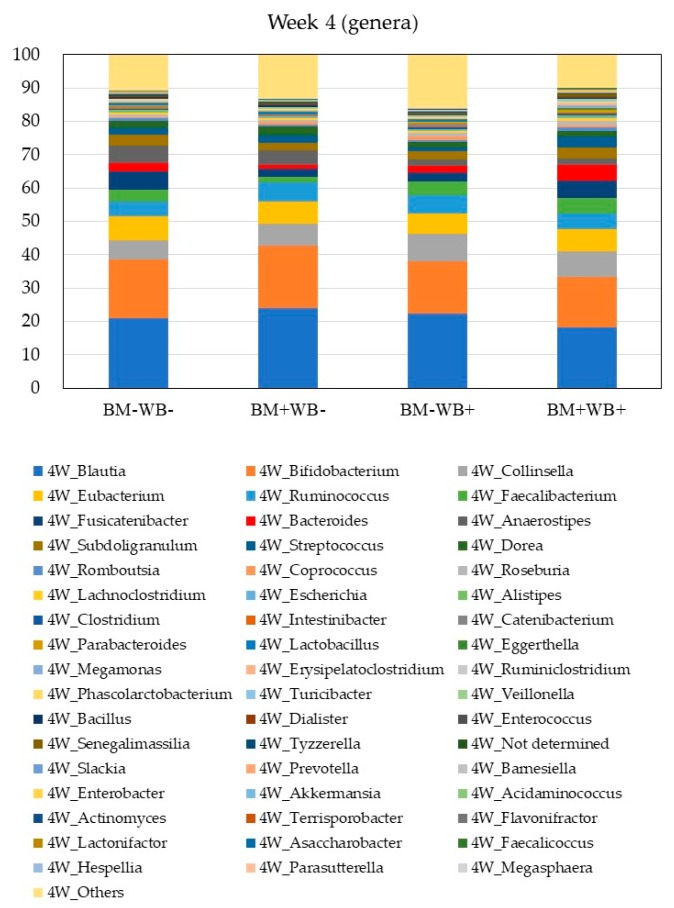
The average relative abundances of detected bacteria genera in stool samples obtained at week 4. A significant interactive effect of WB and BM on the abundances of *Bacteroides* was observed (*p* < 0.05). The abundances of *Anaerostipes* was significantly lower in the WB+ groups than in the WB− groups at week 4 (*p* < 0.05).

The relative abundances of bacterial phyla in the fecal samples of participants at baseline and week 4 are shown in Table 5 and Figure 3 and Figure 4. A significant interactive effect of WB and BM on the abundance of *Bacteroidetes* were observed at week 4, whereas no significant differences among groups were observed at baseline. There were no significant differences in the abundances of other bacterial phyla. Changes in the relative abundances of bacterial phyla in the fecal samples of participants between baseline and week 4 are shown in Table S4. There were no significant differences in the abundances of other bacterial phyla. Significant changes in the relative abundances of bacterial phyla between baseline and week 4 were not observed.

The relative abundances of selected bacterial genera in the fecal samples of participants at baseline and week 4 are shown in Table 6. A significant interactive effect of WB and BM on the abundance of *Bacteroides* was observed at week 4, whereas no significant differences were observed at baseline. The abundances of most butyrate-producing genera including *Ruminococcus, Faecalibacterium, Coprococcus, Roseburia*, and *Ruminiclostridium*, but excluding *Anaerostipes*, at week 4 were significantly higher in the WB groups (WB+BM− and WB+BM+) than in the non-WB groups (WB−BM− and WB−BM+), whereas no significant differences were observed at baseline. The abundances of most butyrate-producing genera at baseline were significantly lower in the BM groups (WB−BM+ and WB+BM+) than in the non-BM groups (WB−BM− and WB+BM−), whereas significant differences were not observed at week 4. By contrast, the abundance of *Anaerostipes* was significantly lower in the WB groups (WB+BM− and WB+BM+) than in the non-WB groups (WB−BM− and WB−BM+). There were no significant differences in other bacteria genera. Changes in the relative abundances of bacterial genera in the fecal samples of participants between baseline and week 4 are shown in Table S5. A significant interactive effect of WB and BM on the abundance of genus *Anaerostipes* was observed. By contrast, changes in the abundance of *Clostridium* were significantly more pronounced in the BM groups (WB−BM+ and WB+BM+) than in the non-BM groups (WB−BM− and WB+BM−). The same tendency was observed for the abundances of most butyrate-producing genera (*p* = 0.060). There were no significant changes between baseline and week 4 in other bacteria genera.

Comparisons of the diversity of the fecal microbiota between the samples obtained at baseline and those obtained at week 4 are shown in Table 7. No significant differences were observed in any diversity indexes.

### 3.4. Fecal Organic Acids and Putrefaction Products

The data for fecal organic acids and putrefaction products from one individual in the WB−BM+ group was excluded based on outlier detection using the Smirnov–Grubbs test. This anomalous result might have been caused by an error in the fecal sampling procedure. The fecal SCFAs and organic acid concentrations of subjects at baseline and week 4 are shown in Table 8. The concentrations of acetate, *n*-butyrate, and total SCFAs were significantly higher in the WB groups (WB+BM− and WB+BM+) than in the non-WB groups (WB−BM− and WB−BM+). Changes in the fecal concentrations of SCFAs and organic acids of subjects between baseline and week 4 are shown in supplementary Table S6. Changes in the concentrations of acetate and total SCFAs were significantly higher in the WB groups (WB+BM− and WB+BM+) than in the non-WB groups (WB−BM− and WB−BM+). The same tendencies were observed in the concentrations of propionate and *n*-butyrate (*p* = 0.0762 and 0.0566, respectively). There were no significant changes between baseline and week 4 in the concentrations of other SCFAs. 

The concentrations of fecal putrefaction products in the samples obtained at baseline and week 4 are shown in Table 9. The concentrations of *p*-cresol and total putrefaction products except ammonia were significantly lower in the WB groups (WB+BM− and WB+BM+) than in the non-WB groups (WB−BM− and WB−BM+). Changes in the concentrations of fecal putrefaction products between baseline and week 4 are shown in Table S7. There were no significant changes between baseline and week 4 in the concentrations of putrefaction products.

**Figure 5:**
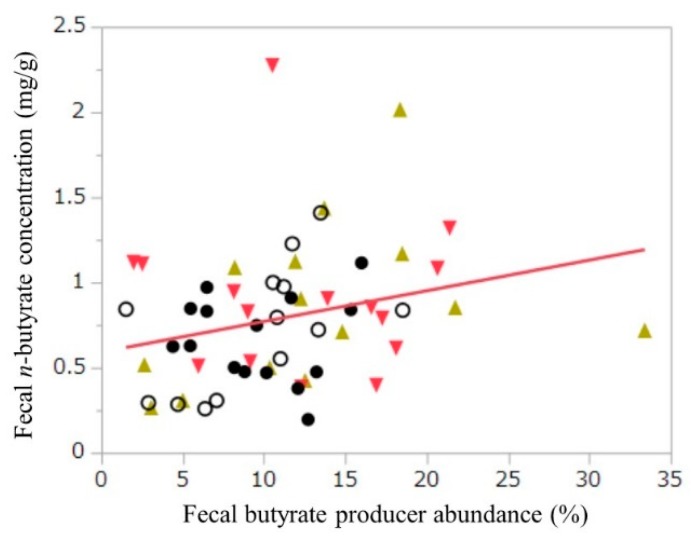
Relationship between the abundance of butyrate producers and the fecal concentration of butyrate at week 4. *r* = 0.32 (Spearman’s rank correlation coefficient), *p* < 0.05. ● WB−BM−, ○ WB−BM+, ▲ WB+BM−, ▼ WB+BM+.

A positive Spearman’s rank moment correlation coefficient was observed between the concentration of *n*-butyrate and the abundance of butyrate-producing bacteria (*p* = 0.012). The relationship between the abundance of butyrate producers and the fecal *n*-butyrate concentration is shown in Figure 5. 

.

## 4. Discussion

In the present work, we studied the effect of dietary supplementation with cereal bars containing WB and/or BM in healthy adult human volunteers. We investigated which dietary fiber source would produce more favorable changes in fecal variables (e.g., higher butyrate, lower phenols, and lower ammonia). We also investigated whether combining WB with BM would modify the fecal microbiota more favorably than supplementation with WB or BM alone. The results indicated that WB increased the fecal concentration of butyrate through increasing the abundance of butyrate-producing bacteria in the gut except for the genus *Anaerostipes*. A previous study examined which bacteria contribute to butyrate production in the gut and concluded that the major butyrate-producing bacteria isolated from the human colon belong to the *Clostridium coccoides (XIVa)* and *Clostridium leptum (IV)* clusters [37]. In our study, we mainly detected butyrate producers in the *Clostridium XIVa* cluster, such as *Roseburia, Anaerostipes,* and *Coprococcus,* and in the *Clostridium IV* cluster, such as *Faecalibacterium*. Amylase-pretreated WB as the sole added energy source was previously reported to be well fermented by butyrate producers in human intestinal microbial communities in anaerobic fermentors [14]. In an experiment using weaned male piglets, the intake of WB increased the cecal concentrations of acetate and butyrate [13]. These findings are consistent with our results. Arabinoxylan, which is an almost-insoluble fiber linked with ferulic acid, is a main component of dietary fiber in WB [9]. It was reported that the release of ferulic acid via human bacterial degradation promoted arabinoxylan solubilization and degradation [14]. Degradation products of arabinoxylan, such as arabinoxylan oligosaccharides, were reported to increase fecal butyrate concentrations and lower the urinary excretion of phenols and *p*-cresol [38]. In our study, with both of WB+BM− and WB+BM+, the daily intake of arabinoxylan from WB was 2.1 g/day, insoluble dietary fiber was 4.8 g/day, and total dietary fiber was 5.1 g/day. In a prior study, the intake of bread containing 2.14 g/day arabinoxylan oligosaccharides increased the fecal concentrations of butyrate and total SCFAs [38]. The intake of more than 2.1 g/day arabinoxylan from WB might effectively increased the fecal concentration of butyrate by increasing the abundance of butyrate producers in the gut. Further intervention studies are needed to elucidate the adequate dose of arabinoxylan.

It has been reported that butyrate exerted a preventive effect against cancer of the colon by promoting cell differentiation, cell-cycle arrest, and apoptosis of transformed colonocytes [38]. Butyrate also inhibited the enzyme histone deacetylase and decreased the transformation of primary bile acids to secondary ones as a result of colonic acidification [38]. Therefore, a significant increase in SCFA production, specifically butyrate, caused by WB intake may result in a protective effect against colonic disorders.

Bacteria related to *Anaerostipes caccae* [39] within the *C. coccoides* cluster were shown to be able to convert acetate and lactate into butyrate, in addition to producing butyrate from carbohydrates [14,40]. In our study, the abundance of *Anaerostipes* was decreased by WB intake. The reason for this is unknown, but it may be related to changes in other bacterial populations such as those of the major butyrate producers *Faecalibacterium* and *Roseburia*.

No diversity indices were affected by WB and BM intake. This finding is consistent with a recent systematic review and meta-analysis that concluded that fiber intervention has no significant effect on α-diversity [41]. Although a lack of fermentable fiber intake decreases diversity indices, an increase in fermentable fiber intake may not change diversity indices in healthy subjects who already consume some fermentable fiber in their staple diets before supplementation.

The abundances of most butyrate-producing genera at baseline were significantly lower in the BM groups than in the non-BM groups, whereas significant differences were not observed at week 4.

Changes in the abundance of *Clostridium* were significantly more pronounced in the BM groups than in the non-BM groups, and changes in the abundances of most butyrate-producing genera were also more prominent in the BM groups than in the non-BM groups (although these changes were not statistically significant). Therefore, BM might have affected the abundance of some butyrate-producing genera even though there were no variations in the abundance of most butyrate-producing genera.

The differential effects of WB and BM intake on fecal butyrate concentrations and butyrate producer populations might have been associated with differences between these fiber sources in intestinal transit time and fermentation speed. In a previous study, purified and semi-purified polysaccharides characteristic of cereals were fermented in vitro with a pig fecal inoculum, using the cumulative gas production technique, to examine the kinetics and end-products of fermentation after 48 h [42]. It was shown that barley β-glucan was fermented rapidly if soluble, while insoluble arabinoxylan was fermented more slowly. Fructan is fermented more rapidly than barley β-glucan. Therefore, fructan and β-glucan in BM are fermented rapidly in the proximal colon and SCFAs might be absorbed at that site. The fructan content in the BM material was 9.0 g/100 g, but the fructan content in the test foods was not analyzed. The contribution of fructan in BM to the changes observed in microbiota and fecal metabolites was unknown in this study. It is important to note that fecal SCFA concentrations are the net result of production and absorption, and it is therefore difficult to evaluate the total SCFA production. The acceleration of the whole-gut transit time by WB may also have contributed to the increase in the concentration of fecal SCFAs in the WB groups [43]. Human studies have indicated that SCFA production is increased with a short transit time, while longer transit times are associated with a shift from carbohydrate to protein fermentation [44,45]. By contrast, the concentrations of *p*-cresol and total putrefaction products except ammonia were significantly lower in the WB groups than in the non-WB groups. The generation of putrefaction products might have been inhibited by a decrease in fecal pH owing to an increase in the concentration of SCFAs. However, the concentration of SCFAs was relatively low at week 4 as compared to baseline. Increases in fecal bulk and water content caused by insoluble dietary fiber intake might have diluted the fecal concentration of SCFAs. 

A significant interactive effect of WB and BM on the abundances of phylum *Bacteroidetes* and genus *Bacteroides* was observed at week 4. *Bacteroidetes* and *Firmicutes* are the major bacterial phyla in the colonic microbiota. Significant decreases in the relative proportions of *Bacteroidetes* and *Firmicutes* have been observed in older adults as compared to younger adults [46]. A study on the fecal bacteria in healthy old volunteers living in the local community; old, hospitalized patients; and old, hospitalized patients receiving antibiotic treatment [47] exhibited a decrease in the abundance of the *Bacteroides-Prevotella* group in the old, hospitalized patients. IgA helps to neutralize the toxins produced by microbes and prevents the adherence of the microbiota to the intestinal lumen. The induction of secretory IgA appears to be more efficient in the presence of *Bacteroidetes* [48]. Taken together, the combined effect of WB and BM in increasing the abundance of the phylum *Bacteroidetes* can be expected to be beneficial for preventing age-related colonic disorders.

There are several potential limitations of our study that may partly explain the lack of an observed effect of the combination of WB and BM on the concentrations of fecal metabolites. First, the relatively small sample size for each arm may have contributed to the observed non-significant results. Secondly, the relatively low β-glucan intake from BM was 0.7 g/day with both of WB−BM+ and WB+BM+, and may not have been sufficient to cause a significant increase in fecal metabolite concentrations. Finally, the intervention period was relatively short. Longer intervention studies may be needed to elucidate the effects of the combination of WB and BM on colonic health.

## 5. Conclusions

The consumption of whole grains has been proposed to improve colonic health. In this study, we investigated the influence of the dietary fiber sources WB and BM on fecal microbiota composition, SCFA production, and fecal putrefaction products in healthy Japanese adults. WB was associated with elevated concentrations of fecal SCFAs including butyrate owing to an increase in the abundance of butyrate-producing bacteria in the colon. Additionally, the combination of WB and BM was associated with an altered composition of the microbiota, favoring an increase in the abundance of the genus *Bacteroides*.

## Figures and Tables

**Table 1:** Baseline characteristics of subjects.^a^

		WB−BM− Group	WB+BM− Group	WB−BM+ Group	WB+BM+ Group
Subjects	*N*	15	15	15	15
Sex (male/female)	*N*	5/10	5/10	5/10	4/11
Age ^b^	Y	46.4 ± 8.80	46.4 ± 7.9	46.5 ± 8.0	46.4 ± 10.5
Height	Cm	160.6 ± 6.6	162.4 ± 8.6	161.5 ± 6.6	160.3 ± 6.3
Body weight	Kg	52.1 ± 8.8	58.3 ± 8.5	59.7 ± 15.1	53.0 ± 7.1
Body mass index	kg/m^2^	20.1 ± 2.4	22.1 ± 2.4	22.9 ± 5.6	20.6 ± 2.8
Body fat	%	23.1 ± 6.4	27.5 ± 5.9	26.0 ± 9.8	24.9 ± 9.2
Systolic blood pressure	mmHg	114.7 ± 10.7	114.1 ± 11.7	114.5 ± 12.5	112.7 ± 11.9
Diastolic blood pressure	mmHg	74.1 ± 8.0	70.0 ± 8.2	71.6 ± 10.4	72.7 ± 10.1
Pulse rate	bpm	69.2 ± 10.3	65.2 ± 8.1	66.5 ± 8.0	69.7 ± 8.1

WB, wheat bran; BM, BARLEYmax. ^a^ There were no statistical differences among groups on the basis of the Tukey–Kramer and Steel–Dwass multiple comparison tests. ^b^ Mean ± standard deviation (SD).

**Table 2:** Components of test foods.

	WB−BM−	WB+BM−	WB−BM+	WB+BM+
Cellulose	4.5 g	1.7 g	2.8 g	―
Wheat bran	―	6.0 g	―	6.0 g
BARLEYmax	―	―	6.0 g	6.0 g
Wheat flour	17.9 g	14.9 g	13.8 g	10.6 g
Egg	4.4 g	4.4 g	4.4 g	4.4 g
Margarine	9.2 g	9.2 g	9.2 g	9.2 g
Emulsifier	0.5 g	0.5 g	0.5 g	0.5 g
Brown sugar	7.3 g	7.3 g	7.3 g	7.3 g
Cocoa powder	0.9 g	0.9 g	0.9 g	0.9 g
Salt	0.05 g	0.05 g	0.05 g	0.05 g
Vanilla flavor	1.2 g	1.2 g	1.2 g	1.2 g
Caramel color	0.2 g	―	―	―
Total	46.15g	46.15 g	46.15 g	46.15 g

**Table 3:** Nutritional profiles of test foods. ^a^

	WB−BM−	WB+BM−	WB−BM+	WB+BM+
Energy (kcal/100 g)	454	447	455	448
Protein (g/100 g)	6.0	7.8	7.1	8.6
Fat (g/100 g)	22.1	22.6	22.2	23.4
Available carbohydrate (g/100 g)	50.9	46	49.6	43.5
Total dietary fiber (g/100 g)	12.7	13.0	12.0	12.1
Soluble dietary fiber (g/100 g)	<0.5	1.1	1.2	1.8
Insoluble dietary fiber (g/100 g)	12.0	11.5	10.5	10.7
β-glucan (g/100 g)	nd.	0.4	0.8	1.2
Arabinoxylan (g/100 g) ^b^	nd.	2.6	1.0	3.6
Resistant starch (g/100 g)	nd.	nd.	1.4	1.1

^a^ Weight of test food: 40.7 g after baking process. ^b^ Calculated value from analytical data of arabinoxylan in wheat bran and BARLEYmax.

**Table 4:** Energy and nutrition intakes of subjects during the test period. ^a^

	Group	Average Daily Record	*p*-Value ^b^
Energy (kcal)	WB−BM−	1403.0 ± 374.2	0.207
	WB+BM−	1491.3 ± 446.8	
	WB−BM+	1361.8 ± 320.2	
	WB+BM+	1364.2 ± 361.9	
Protein (g)	WB−BM−	51.7 ± 16.8	0.343
	WB+BM−	54.3 ± 18.2	
	WB−BM+	51.5 ± 13.5	
	WB+BM+	48.9 ± 15.2	
Fat (g)	WB−BM−	51.6 ± 20.7	0.261
	WB+BM−	55.0 ± 26.5	
	WB−BM+	47.6 ± 15.4	
	WB+BM+	49.8 ± 20.0	
Carbohydrate (g)	WB−BM−	174.5 ± 54.9	0.604
	WB+BM−	185.7 ± 57.6	
	WB−BM+	174.4 ± 50.9	
	WB+BM+	175.4 ± 53.6	
Total dietary fiber (g)	WB−BM−	8.8 ± 3.4	0.904
	WB+BM−	8.3 ± 3.3	
	WB−BM+	8.6 ± 3.4	
	WB+BM+	8.6 ± 3.6	

^a^ Average daily records for 4 days. All values are mean ± SD, *n* = 15 in all groups. ^b^ One-way ANOVA.

**Table 5:** Relative abundances of bacteria phyla in fecal samples obtained at baseline and week 4. ^a^

				*p*-Value (Two-Way ANOVA)					
Phylum	Group	Baseline	Week 4	WB		BM		WB × BM	
				Baseline	Week 4	Baseline	Week 4	Baseline	Week 4
*Firmicutes %*	WB−BM−	64.0 ± 8.5	69.9 ± 13.6	0.53	0.79	0.60	0.86	0.73	0.98
	WB+BM−	67.5 ± 14.0	69.0 ± 13.1						
	WB−BM+	63.4 ±16.4	70.7 ± 16.0						
	WB+BM+	64.4 ± 15.2	69.6 ± 14.8						
*Actinobacteria* %	WB−BM−	28.8 ± 12.6	24.5 ± 13.4	0.48	1.00	0.64	0.96	0.75	0.56
	WB+BM−	24.8 ± 13.0	26.7 ± 12.3						
	WB−BM+	29.3 ± 16.0	26.5 ± 15.7						
	WB+BM+	27.8 ± 16.4	24.3 ± 15.4						
*Bacteroidetes* %	WB−BM−	6.0 ± 5.9	4.7 ± 2.7	0.34	0.26	0.50	0.71	0.60	0.02
	WB+BM−	6.7 ± 6.5	3.6 ± 3.5						
	WB−BM+	4.0 ± 4.7	2.0 ± 1.7						
	WB+BM+	6.4 ± 7.7	5.5 ± 6.1						
*Proteobacteria* %	WB−BM−	0.9 ± 1.0	0.6 ± 0.0	0.23	0.59	0.19	0.96	0.38	0.85
	WB+BM−	0.5 ± 0.9	0.5 ± 1.2						
	WB−BM+	3.0 ± 0.0	0.6 ± 1.3						
	WB+BM+	1.0 ± 1.9	0.4 ± 0.4						

^a^ All values are mean ± SD, *n* = 15 in all groups.

**Table 6:** Relative abundances of selected bacteria genera in fecal samples obtained at baseline and week 4. ^a^

				*p*-Value (Two-Way ANOVA)					
Genus	Group	Baseline	Week 4	WB		BM		WB × BM	
				Baseline	Week 4	Baseline	Week 4	Baseline	Week 4
*Bacteroides* %	WB−BM−	4.2 ± 4.8	3.1 ± 1.8	0.34	0.31	0.46	0.87	0.70	0.03
	WB+BM−	4.9 ± 5.7	2.2 ± 2.2						
	WB−BM+	2.7 ± 3.9	1.4 ± 1.3						
	WB+BM+	4.5 ± 5.8	3.7 ± 4.5						
*Bifidobacterium* %	WB−BM−	19.5 ± 10.3	17.2 ± 12.7	0.23	0.77	0.52	0.97	0.50	0.82
	WB+BM−	13.6 ± 10.8	17.0 ± 13.4						
	WB−BM+	19.4 ± 13.1	18.0 ± 10.7						
	WB+BM+	17.7 ± 13.5	16.4 ± 12.5						
*Lactobacillus* %	WB−BM−	0.2 ± 0.4	0.4 ± 1.0	0.55	0.96	0.74	0.33	0.41	0.44
	WB+BM−	0.2 ± 0.6	0.7 ± 2.4						
	WB−BM+	0.4 ± 0.9	0.4 ± 0.7						
	WB+BM+	0.1 ± 0.4	0.1 ± 0.2						
*Prevotella* %	WB−BM−	0.1 ± 0.2	0.2 ± 0.4	0.45	0.38	0.47	0.42	0.58	0.20
	WB+BM−	0.1 ± 0.2	0.1 ± 0.2						
	WB−BM+	0.4 ± 1.5	0.1 ± 0.3						
	WB+BM+	0.1 ± 0.3	0.4 ± 0.9						
*Clostridium* %	WB−BM−	6.8 ± 4.5	4.9 ± 3.4	0.66	0.67	0.55	0.35	0.06	0.36
	WB+BM−	4.4 ± 3.3	3.6 ± 2.9						
	WB−BM+	4.2 ± 3.8	5.0 ± 4.1						
	WB+BM+	5.7 ± 4.6	5.5 ± 5.0						
*Anaerostipes* %	WB−BM−	3.5 ± 2.5	5.2 ± 5.3	0.97	<0.01	0.29	0.71	0.14	0.69
	WB+BM−	2.0 ± 2.3	2.2 ± 2.2						
	WB−BM+	3.1 ±3.6	4.5 ± 4.3						
	WB+BM+	4.7 ± 6.2	2.2 ± 1.8						
Butyrate producers ^b^ %	WB−BM−	12.7 ± 7.4	8.7 ± 4.4	0.77	0.05	0.03	0.76	0.22	0.80
	WB+BM−	11.1 ± 6.4	12.0 ± 7.1						
	WB−BM+	6.9 ± 6.5	8.6 ± 4.5						
	WB+BM+	9.5 ± 5.7	11.2 ± 5.4						

^a^ All values are mean ± SD, *n* = 15 in all groups. ^b^ Sum of *Ruminococcus*, *Faecalibacterium*, *Coprococcus*, *Roseburia*, and *Ruminiclostridium*.

**Table 7:** Comparison of gut microbiota diversity between fecal samples obtained at baseline and at week 4. ^a^

				*p*-Value (Two-Way ANOVA)					
Diversity Index	Group	Baseline	Week 4	WB		BM		WB × BM	
				Baseline	Week 4	Baseline	Week 4	Baseline	Week 4
Phylogenetic diversity (PD_whole_tree)	WB−BM−	27.9 ± 4.3	27.1 ± 6.3	0.38	0.93	0.54	0.91	0.70	0.81
	WB+BM−	28.9 ± 10.0	27.4 ± 7.5						
	WB−BM+	25.9 ± 8.5	27.4 ± 7.8						
	WB+BM+	28.5 ± 6.5	26.8 ± 6.0						
Chao1	WB−BM−	1123.3 ± 184.5	1144.4 ± 294.7	0.24	0.90	0.34	0.62	0.96	0.89
	WB+BM−	1230.4 ± 451.4	1166.4 ± 322.4						
	WB−BM+	1044.8 ± 348.8	1115.6 ± 332.7						
	WB+BM+	1142.9 ± 286.3	1114.1 ± 300.9						
Observed number of OTUs (observed_species)	WB−BM−	630.4 ± 101.6	626.5 ± 149.6	0.20	0.74	0.35	0.89	0.98	0.73
	WB+BM−	688.7 ± 251.4	655.0 ± 165.4						
	WB−BM+	585.8 ± 180.7	635.4 ± 175.8						
	WB+BM+	646.4 ± 150.8	634.8 ± 167.5						
Shannon index	WB−BM−	5.5 ± 0.5	5.6 ± 0.5	0.15	0.70	0.39	0.79	0.45	0.28
	WB+BM−	5.6 ± 0.7	5.5 ± 0.5						
	WB−BM+	5.2 ± 0.7	5.4 ± 0.5						
	WB+BM+	5.6 ± 0.6	5.7 ± 0.7						

^a^ All values are mean ± SD, *n* = 15 in all groups.

**Table 8:** Fecal SCFA concentrations of subjects at baseline and week 4. ^a^

				*p*-Value (Two-Way ANOVA)					
Phylum	Group	Baseline	Week 4	WB		BM		WB × BM	
				Baseline	Week 4	Baseline	Week 4	Baseline	Week 4
Acetate (mg/g feces)	WB−BM−	2.18 ± 1.42	1.96 ± 1.10	0.60	0.04	0.42	0.39	0.35	0.90
	WB+BM−	2.30 ± 0.88	2.48 ± 0.98						
	WB−BM+	2.73 ± 1.17	2.16 ± 0.97						
	WB+BM+	2.26 ± 1.19	2.74 ± 0.94						
Propionate (mg/g feces)	WB−BM−	1.09 ± 0.59	0.90 ± 0.37	0.55	0.16	0.80	0.70	0.90	0.43
	WB+BM−	1.02 ± 0.47	1.16 ± 0.66						
	WB−BM+	1.14 ± 0.40	0.95 ± 0.31						
	WB+BM+	1.04 ± 0.57	1.02 ± 0.39						
*iso*-Butyrate (mg/g feces)	WB−BM−	0.13 ± 0.08	0.12 ± 0.06	0.60	0.66	0.24	0.89	0.77	0.88
	WB+BM−	0.11 ± 0.08	0.12 ± 0.07						
	WB−BM+	0.15 ± 0.11	0.13 ± 0.07						
	WB+BM+	0.15 ± 0.09	0.12 ± 0.06						
*n*-Butyrate (mg/g feces)	WB−BM−	0.89 ± 0.86	0.67 ± 0.25	0.53	0.05	0.98	0.93	0.97	0.59
	WB+BM−	0.78 ± 0.53	0.96 ± 0.62						
	WB−BM+	0.88 ± 0.47	0.74 ± 0.36						
	WB+BM+	0.78 ± 0.54	0.91 ± 0.47						
Total SCFA (mg/g feces)	WB−BM−	4.64 ± 2.85	3.94 ± 1.45	0.53	0.04	0.50	0.58	0.67	0.81
	WB+BM−	4.52 ± 1.85	4.98 ± 2.03						
	WB−BM+	5.29 ± 1.72	4.29 ± 1.63						
	WB+BM+	4.66 ± 2.32	5.12 ± 1.61						

^a^ All values are mean ± SD, *n* = 15 in all groups except *n* = 14 in the WB−BM+ group.

**Table 9:** Concentrations of fecal putrefaction products in stool samples obtained at baseline and week 4. ^a^

Putrefaction Product				*p*-Value (Two-Way ANOVA)					
	Group	Baseline	Week 4	WB		BM		WB × BM	
				Baseline	Week 4	Baseline	Week 4	Baseline	Week 4
Ammonium (mg/g)	WB−BM−	0.5 ± 0.2	0.5 ± 0.2	0.777	0.872	0.568	0.701	0.367	0.892
	WB+BM−	0.4 ± 0.2	0.5 ± 0.2						
	WB−BM+	0.5 ± 0.3	0.5 ± 0.2						
	WB+BM+	0.5 ± 0.4	0.4 ± 0.2						
*p*-Cresol (μg/g)	WB−BM−	75.8 ± 67.4	59.6 ± 57.3	0.129	0.043	0.191	0.284	0.616	0.416
	WB+BM−	53.7 ± 58.9	41.8 ± 51.2						
	WB−BM+	113.5 ± 110.1	86.1 ± 60.9						
	WB+BM+	70.7 ± 72.3	45.6 ± 42.0						
Indole (μg/g)	WB−BM−	46.7 ± 30.8	33.7 ± 23.4	0.040	0.070	0.459	0.546	0.503	0.720
	WB+BM−	25.6 ± 19.8	21.6 ± 12.4						
	WB−BM+	47.1 ± 39.5	35.0 ± 24.0						
	WB+BM+	36.3 ± 22.9	26.8 ± 22.6						
Total putrefaction products (except ammonium) (μg/g)	WB−BM−	134.4 ± 92.1	102.2 ± 67.9	0.604	0.037	0.421	0.385	0.346	0.896
	WB+BM−	89.6 ± 74.1	72.9 ± 63.8						
	WB−BM+	174.6 ± 161.4	127.5 ± 83.7						
	WB+BM+	121.4 ± 91.0	81.5 ± 50.0						

^a^ All values are mean ± SD, *n* = 15 in all groups except *n* = 14 in the WB−BM+ group.

## Supplementary Materials

The following are available online at http://www.mdpi.com/2072-6643/10/12/1980/s1. Supplementary Table S1: Effects of wheat bran and BARLEYmax intake on body weight and body mass index, body fat, and blood pressure at baseline and after 4 weeks. Supplementary Table S2: Effects of wheat bran and BARLEYmax on serum biochemistry at baseline and after 4 weeks. Supplementary Table S3: Effects of wheat bran and BARLEYmax on blood markers. Supplementary Table S4: Changes in relative abundances of bacteria phyla in fecal samples between baseline and week 4. Supplementary Table S5: Changes in relative abundances of selected bacteria genera in fecal samples between baseline and week 4. Supplementary Table S6: Changes in fecal SCFA and organic acid concentrations of subjects between baseline and week 4. Supplementary Table S7: Changes in concentrations of fecal putrefaction products in stool samples between baseline and week 4.
